# Evaluation of labor and childbirth care by nurse-midwives in Brazilian private hospitals: results of a quality improvement project

**DOI:** 10.1186/s12978-022-01537-0

**Published:** 2023-01-19

**Authors:** Fabrine C. Souza, Rosa Maria Soares Madeira Domingues, Jacqueline Alves Torres, Maysa Luduvice Gomes, Maria do Carmo Leal

**Affiliations:** 1 Programa de pós-graduação em epidemiologia em saúde pública, Escola Nacional de Saúde Pública Sérgio Arouca, Fundação Oswaldo Cruz, Rua Leopoldo Bulhões, 1480 - Manguinhos, Rio de Janeiro, RJ 21041-210 Brazil; 2 Laboratório de Pesquisa Clínica em DST/Aids, Instituto Nacional de Infectologia Evandro Chagas, Fundação Oswaldo Cruz, Rio de Janeiro, Brazil; 3 Institute for Healthcare Improvement - IHI, Boston, MA USA; 4 Faculdade de Enfermagem, Departamento de Enfermagem Materno-Infantil, Universidade do Estado do Rio de Janeiro, Rio de Janeiro, Brazil; 5 Escola Nacional de Saúde Pública Sérgio Arouca, Fundação Oswaldo Cruz, Rio de Janeiro, Brazil

**Keywords:** Health evaluation, Hospitals, Private, Midwifery, Quality improvement, Natural childbirth

## Abstract

**Background:**

In 2015, a quality improvement project—the “Adequate Childbirth Project” (*Projeto Parto Adequado*, or PPA)—was implemented in Brazilian private hospitals with the goal of reducing unnecessary cesarean sections. One of the strategies adopted by the PPA was the implementation of labor and childbirth care by nurse-midwives. The objective of this study is to evaluate the results of the PPA in the implementation and adequacy of labor and childbirth care by nurse-midwives in Brazilian private hospitals.

**Methods:**

Cross-sectional, hospital-based study, carried out in 2017, in 12 hospitals participating in the PPA. We assessed the proportion of women assisted by nurse-midwives during labor and childbirth care and the adequacy of 13 care practices following parameters of the World Health Organization. Women assisted in the PPA model of care and in the standard of care model were compared using the chi-square statistical test.

**Results:**

4798 women were interviewed. Women in the PPA model of care had a higher proportion of labor (53% × 24.2%, p value < 0.001) and vaginal birth (32.7% × 11.3%, p value < 0.001), but no significant differences were observed in the proportion of women assisted by nurse-midwives during labor (54.8% × 50.1%, p value = 0.191) and vaginal birth (2.2% × 0.7%, p value = 0.142). The implementation of recommended practices was adequate, except the use of epidural analgesia for pain relief, which was intermediate. There was a greater use of recommended practices including “oral fluid and food”, “maternal mobility and position”, “monitoring of labor”, “use of non-pharmacological methods for pain relief” and “epidural analgesia for pain relief” in women assisted by nurse-midwives in relation to those assisted only by doctors. Many non-recommended practices were frequently used during labor by nurse-midwives and doctors.

**Conclusions:**

There was an increase in the proportion of women with labor and vaginal birth in the PPA model of care and an appropriate use of recommended practices in women assisted by nurse-midwives. However, there was no difference in the proportion of women assisted by nurse-midwives in the two models of care. The expansion of nursing participation and the reduction of overused practices remain challenges.

## Plain Language Summary

Brazil has a high rate of cesarean sections with low participation of nurse-midwives in childbirth care. Nurse-midwife care is associated with less use of interventions, lower proportions of cesarean sections and greater women’s satisfaction. In 2015, a quality improvement project, called the “Adequate Childbirth Project” (*Projeto Parto Adequado*, PPA), was implemented in Brazilian private hospitals to reduce unnecessary cesarean sections. One of its components is the expansion of nurse-midwives in labor and childbirth care. In this study, we evaluated the results of PPA on childbirth care by nurse-midwives. Data were collected in 12 hospitals with interviews with 4798 women. Women assisted in the PPA model of care had a higher proportion of labor and vaginal birth, but there was no increase in the proportion of women assisted by nurse-midwives, neither during labor nor vaginal birth. The use of recommended practices was adequate, except for the use of epidural analgesia, with greater use of some recommended practices in women assisted by nurse-midwives compared to those seen only by doctors. However, we observed excessive use of non-recommended practices during labor, both by doctors and nurses. We can conclude that there was an increase in the proportion of women with labor and vaginal birth in the PPA model of care and a greater use of recommended practices in women assisted by nurse-midwives, but without an increase in the proportion of women assisted by nurses. The expansion of nurse-midwives’ participation in childbirth care and the reduction of non-recommended practices therefore continue to be challenges.

## Introduction

Brazil has almost universal coverage of hospital childbirth care, with a model of care characterized by the excessive use of interventions [1–3]. Cesarean-section (CS) rates have increased systematically in the country since the 1970s, and today more than half of the live-births in Brazil occur by means of CS [4]. Considering the characteristics of the population that can affect the cesarean section rate, especially the number of women with previous cesarean sections [5], the adjusted reference rate for the Brazilian population would be 25–30%, which therefore is half of the current values observed [6, 7].

CS rates differ between the public and private sectors, reaching almost 90% of childbirths in the latter, being determined not only by clinical factors, but by social and economic determinants and by the organization of obstetric care services [3, 8]. In the private sector, prenatal and childbirth care is usually provided by just one doctor of the woman’s choice, with the possibility of scheduling a cesarean section, according to her wishes and/or the obstetrician’s recommendation [3, 9–11]. The participation of nurse-midwives and midwives in childbirth care in this sector is very low, despite current national legislation, which legally enables nurses to provide prenatal and childbirth care, and national and international recommendations to include these professionals in labor and childbirth care [2, 12–15].

The National Supplementary Health Agency (*Agência Nacional de Saúde Suplementar*, or ANS), a regulatory agency linked to the Brazilian Ministry of Health that is responsible for the health insurance sector in Brazil [16], has been developing systematic actions to improve the quality of obstetric and neonatal care and to reduce the proportion of cesarean sections in the private health sector [16]. One of these actions is the “Adequate Childbirth Project” (*Projeto Parto Adequado* or PPA), a quality improvement project that aims to identify innovative and viable models of childbirth care that value vaginal birth and reduce the proportion of cesarean sections without clinical indication. This initiative also aims to offer women and babies efficient care throughout pregnancy, labor, childbirth and postpartum, considering the hospital structure, training of the multidisciplinary team, evidence-based medicine and socio-cultural-emotional conditions of a pregnant woman and her family [16].

The PPA quality improvement project has four components: governance, women’s participation, reorganization of the model of care and monitoring of indicators [17]. In each of the components, several activities were defined to be implemented by the hospitals that joined the program. One of the activities listed in the “reorganization of the model of care” component is the expansion of labor and vaginal birth care by nurse-midwives and midwives, based on scientific evidence that demonstrates greater satisfaction of women and lower rates of interventions, especially of cesarean sections without clinical indication, in models of care involving these professionals [14, 18–20].

Women assisted by nurse midwives and midwives are more likely to have a spontaneous vaginal delivery, have fewer episiotomy, instrumental delivery, suffer less miscarriage, neonatal death or premature delivery. In contrast, there is no greater likelihood of adverse outcomes for women and their babies when assisted by nurses and midwives. Maternal satisfaction with information, advice, explanations, place of delivery, preparation for childbirth, pain relief and behavior of professionals are also present in the evaluation of care, showing positive results in this care model [14].

The collaborative model of care, characterized by the integration of a physician and a nurse-midwife in the labor and childbirth care team, was proposed. In this collaborative model, a nurse-midwife is responsible for the integral assistance to women of habitual risk, ensuring the possibility of immediate referral to the obstetrician in cases of complications; assistance to high-risk women is provided in partnership with the obstetrician, due to the increased risk of clinical and/or obstetric complications during labor and childbirth [20].

The PPA was structured in three phases. Phase 1, developed between 2015 and 2016, aimed to test the intervention and had the participation of 35 public and private hospitals and 19 health insurance companies. Phase 2, which started in 2017 and is still in progress, is characterized by extending the project to a variety of healthcare providers and companies. Finally, Phase 3, launched in October 2019, aims to disseminate strategies to improve the quality of childbirth care on a large scale, with the possibility of including all maternities and health insurance companies in Brazil [16, 17].

After the implementation of the PPA, an evaluative study called “Healthy Birth” was conducted, with the objective of assessing the degree of implementation of the PPA activities, evaluating the variation of its effects, comparing the effectiveness of the different models of childbirth care, assessing the cost-effectiveness of the PPA and identifying barriers and facilitators for the implementation of the project [21]. Using data from the “Healthy Birth” research, the objective of this study is to evaluate the results of the PPA in the implementation and adequacy of labor and childbirth care provided by nurse-midwives in Brazilian private hospitals.

## Methods

The “Healthy Birth” study is an evaluative, hospital-based study with a sectional design that was carried out between March and August 2017, six to eight months after the end of the implementation of the first phase of the PPA. Twelve private hospitals participated in this study, and they were selected intentionally from the 35 hospitals (23 private and 12 public) that participated in phase 1 of the PPA. These 12 hospitals were located in three macro-regions of Brazil (South, Southeast and Northeast). The selection criteria for hospitals, as well as the sample size calculation, inclusion and exclusion criteria for women, data collection and training of the research team are described in Torres et al. [21]. In summary, approximately 400 women were included in each of the 12 hospitals selected for the study. Face-to-face interviews were carried out in the first 24 h after vaginal birth or CS and information was extracted from medical records after hospital discharge. Eligible women were included sequentially until completing the planned sample in each hospital.

In this article, our exposure variable was the model of care: the model of care recommended by the PPA, which we call the “PPA model of care”, and the model of care currently in place in private hospitals, which we call the “standard of care model”. The outcome variables were the proportion of women who had spontaneous or induced labor, the proportion of women assisted in collaborative work of doctors and nurse-midwives, the proportion of vaginal births, the proportion of vaginal births assisted by nurse-midwives, and the adequacy of care provided during labor, using the World Health Organization (WHO) recommendations as a parameter [22, 23].

Women in the “PPA model of care” would be exposed to the activities recommended by the quality improvement project, which includes access to information during pregnancy, visits to the maternity hospital, preparation of the birth plan by the pregnant woman, assistance during labor and childbirth care by doctors and nurse-midwives working collaboratively, and use of evidence-based practices. Women in the “Standard model of care” would be assisted according to current practice in Brazilian private hospitals, characterized by labor and childbirth care by the same doctor who performs prenatal care, low participation of nurse-midwives, high proportion of antepartum cesarean section and intensive use of interventions in labor and childbirth care [8, 13, 15, 21].

In the first phase of the PPA, each hospital defined the target population to be exposed to the quality improvement project. In two hospitals the target population of the PPA consisted of all primiparous women, in two hospitals it consisted of women in Robson Groups [24] 1 to 4 and in eight hospitals it consisted of women admitted by the hospital’s staff on duty (one of which was limited to women in groups 1 to 4 of the Robson Ten Group Classification System and another to women without an anterior uterine scar).

The classification of women in the “PPA model of care” and in the “Standard of care model” was made based on information obtained in the interview with the puerperal woman—for the identification of women assisted by the hospital team on duty or by external teams—and by medical record data, for identification of primiparous women and classification according to the Robson Group system.

In the first stage of data analysis, we describe and compare the characteristics of women in the “PPA model of care” and in the “Standard of care model”. We verified demographic (age, self-reported skin color), socioeconomic (education; economic class [25], where women in class “A” represent those of the highest economic level; marital status; paying jobs) and obstetric (risk pregnancy, classification according to the Robson Group system) characteristics. It was considered a risky pregnancy when a woman had one of the following conditions during pregnancy: hypertensive syndromes (chronic hypertension, hypertension during pregnancy, pre-eclampsia, eclampsia, HELLP syndrome), gestational and non-gestational diabetes, diagnosis of infection at admission hospital, placenta previa, placental abruption, chronic heart, or liver or kidney disease.

In the second stage of the data analysis, we describe the proportion of women assisted in the “PPA model of care” and in the “Standard of care model” in each hospital, as well as the proportion of women who had spontaneous or induced labor, the proportion of women assisted in collaborative work of doctors and nurse-midwives, the proportion of vaginal births, and the proportion of vaginal births assisted by nurse-midwives according to the model of care.

For calculating the proportion of women assisted in collaborative work, we used the number of women assisted during labor by nurse-midwives, in isolation and/or in collaboration with the medical team, as a numerator in relation to the total number of women who had spontaneous or induced labor. To calculate the proportion of women with vaginal birth assisted by nurse-midwives, we used the number of women with vaginal birth assisted by nurses as a numerator in relation to the total number of women with vaginal birth. For both indicators, we used hospital records as the source of information.

In the third stage of the analysis, we estimated the adequacy of assistance during labor, with the respective 95% confidence intervals (95% CI), using the World Health Organization recommendations as a parameter [22, 23]. The following practices were assessed:

1. Recommended practices: companionship during labor, oral fluids and food, maternal position and mobility, monitoring of labor progression (digital vaginal examination and auscultation of fetal heart rate), non-pharmacological pain relief methods, respect for a woman’s birth plan as a proxy of respectful maternity care, and epidural analgesia for pain relief; and
2. Non-recommended practices: routine intravenous fluid, routine amniotomy, enema on admission, perineal/pubic shaving, cardiotocography during labor in healthy pregnant women with spontaneous labor, and use of oxytocin for prevention of delay in labor in women receiving epidural analgesia.

The following parameters were used to assess the adequacy of recommended practices during labor: “satisfactory”, if ≥ 75%; “Intermediate”, if in the range of 50–74%; “Unsatisfactory”, if in the range of 25–49%; and “very unsatisfactory”, if less than 25%. In the case of non-recommended practices, the parameters used were: “very unsatisfactory”, if ≥ 75%; “Unsatisfactory”, if in the range of 50–74%; “Intermediate”, if in the range of 25–49%; and “satisfactory”, if less than 25% [26]. Due to the small number of vaginal births assisted by nurses, it was not possible to assess the adequacy of practices during vaginal birth care.

Finally, we compared the care practices during labor in women assisted in collaborative work in the “PPA model of care” to those assisted in collaborative work in the “standard of care model”, as well as the care practices used in women in the “PPA model of care” comparing women assisted in collaborative work to those assisted only by doctors.

In the bivariate analyses, we verified differences between proportions using the chi-square test with a significance level of 0.05. This study used a complex sample with the selection of hospitals and later of women. In addition, the sample of women per hospital was fixed, although the hospitals presented different numbers of admissions for childbirth. For these reasons, data weighting and design effect were used throughout the analysis. We used the statistical program SPSS version 17 [27].

## Results

A total of 4798 women were interviewed, 53.6% of whom were assisted in the “PPA model of care” and 46.4% in the “Standard of care model”. Women in the “Standard of care model” were older, belonged to higher economic classes, more frequently lived with a partner, and had higher-risk pregnancies, while women in the “PPA model of care” more often had paying jobs. Most women in the “Standard of care model” belonged to group 5 of the Robson Groups (multiparous women, with a single, cephalic and term pregnancy with a previous uterine scar), while in the “PPA model of care” Group 2 (primiparous women, with a single, cephalic, term pregnancy and induced labor or antepartum CS) was the most frequent. There were no significant differences for the variables of self-reported skin color and years of schooling (Table 1).

**Table 1:** Women’s characteristics according to the type of model of care in private hospitals, Brazil, 2017

Characteristics of women	“PPA model of care” (N = 2571)			“Standard of care model” (N = 2227)			p-value*
	n	%	CI 95%	n	%	CI 95%	
Age							
< 20 years	67	2.6	(2.0;3.3)	16	0.7	(0.5;1.1)	< 0.001
20 to 34 years	1753	68.2	(65.9;70.4)	1315	59.0	(56.7;61.4)	
35 years or older	750	29.2	(27.1;31.4)	896	40.3	(37.9;42.6)	
Self-reported skin color							
White	1733	67.4	(65.4;69.4)	1506	67.7	(65.7; 69.7)	0.249
Black	136	5.3	(4.4;6.3)	93	4.2	(3.4;5.1)	
Mixed	622	24.2	(22.4;26.0)	572	25.7	(24.0;27.6)	
Asian	77	3.0	(2.2;4.1)	52	2.3	(1.6;3.3)	
Indigenous	3	0.1	(0.0;0.3)	1	0.1	(0.0;0.2)	
Education							
Elementary School	117	4.6	(3.9;5.5)	87	3.9	(3.2;4.7)	0.563
High school	997	39.0	(37.0;41.1)	862	38.8	(36.7;41.0)	
University education	738	28.9	(26.7; 31.1)	677	30.5	(28.3;32.7)	
Postgraduate studies	702	27.5	(25.4;29.7)	596	26.8	(24.7;29.0)	
Economic class							
Class A	674	26.2	(24.2;28.3)	698	31.4	(29.2;33.6)	< 0.001
Class B	1414	55.0	(52.7;57.3)	1200	53.9	(51.5;56.2)	
Class C	473	18.4	(16.9;20.0)	324	14.6	(13.2;16.0)	
Class D	9	0.4	(0.2;0.6)	5	0.2	(0.1;0.5)	
Lives with a partner	2352	92.0	(90.7;93.2)	2123	95.6	(94.7;96.4)	< 0.001
Paying jobs	2075	81.2	(79.4;82.9)	1718	77.3	(75.3;79.2)	0.004
Pregnancy complications	514	20.0	(18.2;21.9)	532	23.9	(22.0;25.9)	0.005
Robson classification							
Group 1 (nulliparous, single fetus, cephalic, ≥ 37 weeks and spontaneous labor)	718	27.9	(25.8;30.1)	99	4.4	(3.8;5.2)	< 0.001
Group 2 (nulliparous, single fetus, cephalic, ≥ 37 weeks and induced or cesarean section before labor)	1148	44.6	(42.3;47.0)	355	16.0	(14.8;17.2)	
Group 3 (Multiparous, without previous cesarean section, single fetus, cephalic, ≥ 37 weeks and spontaneous labor)	182	7.1	(6.0;8.3)	68	3.1	(2.4;3.9)	
Group 4 (Multiparous, without previous cesarean section, single fetus, cephalic, ≥ 37 weeks, Induced labor or cesarean section before Labor)	101	3.9	(3.1;5.0)	88	4.0	(3.2;4.8)	
Group 5 (Anterior cesarean section, single fetus, cephalic, ≥ 37 weeks)	146	5.7	(5.0;6.5)	1072	48.2	(45.8;50.5)	
Groups 6 to 9 (nulliparous and multiparous with single and pelvic fetuses; all multiple pregnancies; fetuses in a transverse or oblique situation)	120	4.7	(3.9;5.6)	283	12.7	(11.1;14.5)	
Group 10 (All single gestation, cephalic, < 37 weeks, including previous cesarean section)	156	6.1	(5.2;7.1)	262	11.7	(10.2;13.5)	

*Statistical method used: Pearson's chi-square

The proportion of women in the PPA model of care ranged from 11.2 to 73.4% in the 12 hospitals, with the highest value observed in one of the hospitals where all women assisted by the hospital staff on duty were included in the “PPA model of care”. The proportion of women with spontaneous or induced labour, who were assisted in collaborative work by nurse-midwives and doctors, and who had a vaginal birth varied among hospitals and models of care. Less than 2% of women had a vaginal birth assisted by a nurse. Significant differences between the two models of care were observed for the proportion of women in labor and the proportion of women with vaginal birth, with higher values in the PPA model of care (Table 2).

**Table 2:** Labor and childbirth care in 12 hospitals participating in the “Adequate Chidbirth Project”, Brazil 2017

Hospital	n	Proportion of the women population		Proportion of women with spontaneous or induced labor		Proportion of women assisted during labor by nurse and physician in collaborative work		Proportion of women with vaginal birth		Proportion of women with vaginal birth assisted by nurses	
		PPA model of care	SoC model	PPA model of care	SoC model	PPA model of care	SoC model	PPA model of care	SoC model	PPA model of care	SoC model
1	222	29.7	70.3	63.6	25.8	95.2	87.5	34.8	10.9	4.3	0.0
2	151	11.2	88.8	56.2	19.4	0.0	6.7	31.2	5.9	0.0	0.0
3	287	32.1	67.9	73.9	17.5	1.5	5.9	57.1	5.1	0.0	0.0
4	217	58.3	41.7	60.3	32.2	4.2	4.0	43.7	18.7	1.8	5.9
5	187	45.5	54.5	71.8	18.6	94.8	92.9	54.1	9.8	0.0	0.0
6	561	62.6	37.4	42.0	30.0	2.7	6.4	20.5	16.7	1.4	0.0
7	325	52.0	48.0	64.5	20.5	14.5	11.1	38.1	7.1	0.0	0.0
8	599	56.8	43.2	56.5	27.9	88.2	97.7	38.8	9.7	0.8	0.0
9	323	73.4	26.6	54.7	4.7	68.9	100	32.9	2.3	13.0	0.0
10	324	46.0	54.0	53.3	22.4	44.6	48.1	32.9	10.9	2.0	0.0
11	1098	58.5	41.5	50.9	26.0	63.1	55.4	29.1	10.8	0.0	0.0
12	504	59.0	41.0	41.3	29.5	95.3	81.1	26.8	22.7	3.8	2.1
Total	4798	53.6	46.4	53.0^a^	24.2^a^	54.8^b^	50.1^b^	32.7^a^	11.3^a^	2.2^c^	0.7^c^

SoC model = Standard of care model

Definition of the PPA model of care population: Hospitals 1, 2, 3, 7, 9 and 10 = admission and birth care by hospital team on duty; Hospitals 4 and 8 = Robson groups 1 to 4; Hospital 5 = primiparous and multiparous without previous uterine scar, admitted by hospital team on duty; Hospitals 6 and 12 = primiparous women; Hospital 11 = Robson groups 1 to 4, childbirth care by hospital team on duty

^a^p < 0.001

^b^p = 0.191

^c^p = 0.142, chi-square statistical test

All practices recommended by the WHO [22, 23] had satisfactory performance in women in the “PPA model of care” assisted in collaborative work, except for “epidural analgesia”, which presented intermediate performance. Women assisted in collaborative work in the “Standard of care model” had intermediate performance for “oral fluid and food”, “use of non-pharmacological methods” and “epidural analgesia” (Table 3). Women assisted in collaborative work in the “PPA model of care” had a significantly higher frequency of “companionship during labor” and “monitoring of labor progression” than those assisted in collaborative work in the “Standard of care model”. Regarding non-recommended practices, “enema on admission” and “perineal/pubic shaving” presented satisfactory parameters in both models, being even less frequent in the “PPA model of care”. The performance of “fetal cardiotocography during labor in healthy pregnant women with spontaneous labor” presented an intermediate parameter; while “routine intravenous fluid” and “routine amniotomy” presented unsatisfactory parameters; and “use of oxytocin for prevention of delay in labor in women receiving epidural analgesia”, very unsatisfactory parameters, without significant differences between the two models of care (Table 3).

**Table 3:** Care practices during labor according to the model of care, Brazil, 2017

Care practices	Collaborative work during labor						p value*
	PPA model of care			Standard of care model			
	n	%	CI 95%	n	%	CI 95%	
Recommended practices							
Companionship during labor	593	98.3	(97.1;99.0)	147	94.6	(87.5;97.8)	0.019
Oral fluid and food	397	72.0	(67.2;76.3)	93	65.9	(55.7;74.8)	0.248
Maternal mobility and position	487	93.1	(90.0;95.4)	119	92.6	(85.9;96.3)	0.860
Monitoring of labor progression	407	97.2	(94.5;98.6)	111	91.1	(83.6;95.4)	0.011
Non-pharmacological pain relief	474	73.4	(69.0;77.4)	118	66.1	(57.2;73.9)	0.113
Respected birth plan^a^	108	93.6	(85.9;97.3)	33	92.5	(81.9;97.1)	0.792
Epidural analgesia for pain relief	417	64.6	(60.3;68.7)	98	55.1	(46.2;63.8)	0.055
Non recommended practices							
Routine intravenous fluid	319	49.5	(44.8;54.3)	83	46.4	(37.7;55.4)	0.550
Routine amniotomy^b^	176	64.1	(56.7;70.9)	35	58.4	(42.7;72.6)	0.510
Enema on admission	0	–	–	2	1.1	(0.2;5.1)	0.018
Perineal/pubic shaving	23	3.6	(2.0;6.3)	15	8.5	(4.2;16.2)	0.052
Use of oxytocin for prevention of delay in labor in women receiving epidural analgesia	297	71.2	(65.4;76.3)	72	73.8	(63.0;82.3)	0.652
Cardiotocography during labor in healthy pregnant women with spontaneous labor	118	31.9	(26.3;38.0)	37	30.2	(21.0;41.3)	0.781

Collaborative work during labor = labor assisted by nurse-midwife or nurse-midwife and a doctor

^*^Statistical method used: Pearson's chi-square

^a^Among women who prepared a birth plan (17.9% in the PPA model of care and 20.2% in the standard of care model)

^b^Only in women without spontaneous rupture (n = 60 in the Standard of care model, n = 274 in the PPA model of care)

Among women assisted only by doctors in the “PPA model of care”, the use of “oral fluid and food”, “non-pharmacological pain relief” and “epidural analgesia for pain relief” had intermediate performance, with significantly higher proportion of “oral fluid and food”, “maternal mobility and position”, “monitoring of labor progression”, “non-pharmacological pain relief” and “epidural analgesia for pain relief” in women assisted in collaborative work during labor. There were no significant differences in relation to “companionship during labor” and “respected birth plan” (Table 4). The non-recommended practices were frequent and had no significant difference according to the type of professional that provided assistance during labor, except the use of “enema on admission”, which was satisfactory and used even less frequently in women assisted in collaborative work (Table 4).

**Table 4:** Care practices during labor in women assisted in the PPA model of care, Brazil, 2017

Care practices	Collaborative work during labor			Doctor			p-valor*
	n	%	IC 95%	n	%	IC 95%	
Recommended practices							
Companionship during labor	593	98.3	(97.1;99.0)	476	98.7	(97.5;99.4)	0.530
Oral fluid and food	397	72.0	(67.1;76.3)	250	53.5	(48.4;58.5)	< 0.001
Maternal mobility and position	487	93.1	(90.0;95.4)	329	86.9	(82.5;90.4)	0.008
Monitoring of labor progression	407	97.2	(94.5;98.6)	425	93.5	(90.5;95.6)	0.030
Non-pharmacological pain relief	474	73.4	(69.0;77.4)	332	62.2	(57.4;66.8)	< 0.001
Respected birth plan^a^	108	93.6	(85.9;97.2)	84	97.7	(92.6;99.3)	0.150
Epidural analgesia for pain relief	417	64.6	(60.3;68.8)	298	56.0	(51.5;60.3)	0.007
Non recommended practices							
Routine intravenous fluid	319	49.5	(44.8;54.3)	266	49.9	(45.5;54.3)	0.919
Routine amniotomy^b^	176	64.1	(56.7;70.9)	140	63.1	(56.0;69.8)	0.855
Enema on admission	0	0.0	-	8	1.6	(0.7;3.4)	0.006
Perineal/pubic shaving	23	3.6	(2.0;6.3)	25	4.6	(2.8;7.6)	0.505
Use of oxytocin for prevention of delay in labor in women receiving epidural analgesia	297	71.2	(65.4;76.3)	219	73.7	(67.4;79.1)	0.540
Cardiotocography during labor in healthy pregnant women with spontaneous labor	118	31.9	(26.3;38.1)	106	28.1	(22.9;33.9)	0.358

Collaborative work during labor = labor assisted by a nurse-midwife or nurse-midwife and doctor;

^*^Statistical method used: Pearson's chi-square

^a^Among women who prepared a birth plan (17.9% collaborative work, 16.0% doctors)

^b^Only in women without spontaneous rupture (n = 274 in assistance in collaborative work; n = 222 in assistance only by doctors)

## Discussion

The results of this study show that women assisted in the “PPA model of care”, when compared to the “Standard of care model”, had a significantly higher proportion of induced or spontaneous labor and vaginal birth, but without significant differences in the proportion of women assisted in collaborative work during labor and in the proportion of vaginal births assisted by nurses.

When compared to a national survey on pregnancy and childbirth carried out between 2011 and 2012, there was an increase in the proportion of women assisted in collaborative work during labor in private hospitals, almost twice as much as that observed previously (51.9% vs 28.7%) [28]. In addition, although the proportion of women assisted in collaborative work has not shown a significant difference in the two models of care, in absolute numbers more women benefited from the assistance provided by nurse-midwives in the “PPA model of care”, as the proportion of women in labor in this model of care was higher than in the “Standard of care model”.

The use of recommended practices among women assisted in collaborative work was high in both models of care, which is consistent with the available evidence on childbirth care provided by nurse-midwives [14, 15, 20]. The only practice with intermediate implementation in the “PPA model of care” was the use of epidural analgesia for pain relief, but its evaluation was limited by the lack of data related to the woman's request for analgesia, and it is not possible to verify whether the lower use was due to not requesting analgesia, insufficient provision of this method or both.

However, data prior to the implementation of the PPA quality improvement project are not available, and it cannot be ruled out that the PPA has influenced care more broadly, and not only in the population exposed to the project, increasing the use of recommended practices throughout the entire population of the hospital. In addition, there was a significant difference in the use of some practices, for example, less use of “enema on admission”, and greater presence of “companionship during labor” and “monitoring of labor progression” in the “PPA model of care”. This suggests that the assistance in collaborative work may have been improved by the PPA, resulting in a greater offer of recommended practices, which is already considered satisfactory for most of the women assisted in collaborative work in the “Standard of care model”.

Among all the women in the “PPA model of care” who were assisted either in collaborative work or only by doctors, there was a satisfactory or intermediate use of recommended practices in labor. This is consistent with the results of a study that compared the care provided in hospitals participating in the PPA with private hospitals evaluated in the study “Birth in Brazil”, which showed a significant increase in the use of recommended practices during labor in women assisted in the PPA in hospitals, although not all of them reached a satisfactory level [28]. In the comparison between assistance in collaborative work and that provided only by doctors, there was greater access to “oral fluid and food”, “maternal mobility and position”, “monitoring of labor progression”, and “non-pharmacological methods” and “epidural for pain relief” among women assisted in collaborative work. These results are in line with other national [15, 20] and international [14] studies, where the collaborative work during labor was associated with greater use of recommended practices that can offer greater comfort to women and favor a more positive experience of labor [20].

There was a high use of non-recommended practices regardless of the model of care (PPA or standard of care model) or the professional who provided care during labor (collaborative work or doctors), with only “enema on admission” and “perineal/pubic shaving” showing satisfactory results. Such findings are similar to a previous study that encountered the routine use of practices such as venous infusion and use of amniotomy and oxytocin during labor by both doctors and nurses [15]. However, they differed from a previous study carried out in four public Brazilian hospitals, where the collaborative work was associated with reduced use of amniotomy [20].

The observed result of satisfactory use of recommended practices, although there is still excessive use of non-recommended practices, both in the “PPA model of care” and in the “standard of care model”, suggests a greater facility of incorporating recommended practices than abandoning practices that were once used routinely but are no longer recommended. An English cohort study, which compared maternal and perinatal outcomes and labor interventions according to birthplace, concluded that women assisted in the hospital setting are exposed to more interventions than those who choose other types of birthplace, such as birth centers and home births [29]. Institutional factors such as structure and physical spaces, and rigid protocols, as well as aspects related to the culture and organization of the health system can hinder or facilitate a less interventionist practice by health professionals in general [18, 20, 30].

The higher proportion of vaginal births in the PPA model of care is an important result, but it should be analyzed with caution, as we found that women in the PPA were younger, had lower frequency of pregnancy complications, and most of them belonged to Robson Groups 1 to 3, where lower rates of CS are expected. In the “Standard of care model”, most women belonged to Robson Groups 5 and 2, which are the groups that contribute most to the rate of CS in Brazilian private hospitals [31]. However, other studies suggest that childbirth care practices changed after the implementation of the PPA, and that the increase in the proportion of vaginal births was not the result of a more overall change in the private sector [17, 28].

The proportion of vaginal births assisted by nurse-midwives was very low, only 2.2% in the “PPA model of care”, without a significant difference between the two models of care. Results of a national study carried out between 2011 and 2012 also showed low participation of nurse-midwives in childbirth care: of the 48% of vaginal births in public and private services, only 16.2% were assisted by nurses [15]. The Northern and Southeastern regions recorded the highest frequency of childbirth assistance by nurses, but for different reasons. While in the Northern region the greater participation of nurses is related to the higher occurrence of non-hospital births, which are also assisted by traditional midwives, and the lack of medical professionals, in the Southeastern region the greater role of nursing is due to the implementation of humanization processes of care in the pregnancy-puerperal cycle that has occurred since the 1990s, with the inclusion of nurse-midwives in the childbirth model of care, mainly in public services [4, 13, 15, 32].

More favorable results were found in an evaluation conducted between 2016 and 2017 [33] in Brazilian public hospitals that are part of the “Stork Network” [34], a policy that aims to ensure women the right to reproductive planning and the improvement of humanized care during pregnancy, childbirth and the puerperium in public services of the Brazilian Unified Health System. In this assessment, it was found that more than a third of vaginal births were assisted by nurses, in contrast to the 16.5% found in public maternity hospitals between 2011 and 2012 [15]. Despite these advances, the authors emphasize that the childbirth care provided by nurses is still insufficient, which they attribute to barriers and difficulties for the implementation of childbirth care by nurse-midwives that can be attributed to the still insufficient investment in the training of these professionals, to the low salaries, and to the low hiring rate of nurse-midwives by public hospitals and an even lower one by private hospitals. In addition, the authors point out the obstetricians’ resistance to collaborative work and the disputes expressed by the federal and regional professional councils of medicine.

The low rate of participation of nurse-midwives and midwives in childbirth care in Brazil is the result of historical and social construction, which begins with the medicalization of childbirth in the twentieth century, based on Brazilian medical publications that were essential to increase the visibility of the actions of doctors and to convince the lay public about the effectiveness of medicine [35, 36]. With childbirth becoming an increasingly difficult and risky event, specialized medical assistance became indispensable as a way to identify any variation in normality early and correct its defects [37].

However, more recent studies [38–40] show that in order to ensure efficient and effective care in obstetrics it is necessary that nurse-midwives and midwives are part of the staff within a functional health system [41] which has a qualified health workforce with appropriate skills. This is an important step to ensure that women have access to a quality midwifery service that can provide maternal and newborn health interventions and preventive health care strategies [42].

The expansion of the participation of nurse-midwives and midwives in labor and childbirth care in Brazilian hospitals therefore depends on a sociological and cultural change, deconstructing the notion that a doctor is the only professional trained to monitor pregnancy and childbirth. In addition, there are structural and organizational barriers to overcome [30, 43]. To have a real impact on care, the WHO recommends that countries should have at least one qualified midwife for every 125 births per year [44]. It is estimated that the Brazilian population will grow by 12%, totaling 222.7 million by 2030. Thus, obstetrics services must attend 4.5 million pregnancies per year by 2030, to guarantee universal access to maternal and child care. Currently, 2049 nurse midwives are registered with the Brazilian Nursing Council [45]. Therefore, the low number of available nurse-midwives is in itself a limiting factor for the expansion of childbirth care by nurse-midwives.

Differences in the organization of childbirth care in Brazilian public and private hospitals may also partially explain the best results observed in childbirth care by nurse-midwives in the public sector [1, 46]. This sector presents organization similar to that of many European countries, with childbirth care provided by a professional linked to the hospital who is paid according to the workload, and not to the production of services [1]. In the private sector, prenatal and childbirth care is usually provided by just one doctor of the woman’s choice [32]. This difference in the organization of public and private services plays a major role in the women’s preference for the type of birth [10] and influences the childbirth model of care, marked by the insufficient presence of nurse-midwives and midwives [3].

Finally, in both sectors, the Brazilian model of childbirth care is marked by the overvaluation of technology, a hierarchical system of care, rigid routines, strictly medical responsibility and authority, in addition to the excessive use of clinical interventions and less female protagonism [1, 13, 47, 48]. Such characteristics make it difficult to insert nurse-midwives and midwives in the childbirth scenario, and even more, to respect their autonomy [30, 43].

This study has some limitations. Most of the practices were evaluated based on data from medical records and there is a possibility of differential information bias if the quality of the records varies according to professional category. Not all practices contained in the WHO document have been evaluated due to the lack of available information, which limits the assessment of the care provided. In addition, some practices, such as “monitoring of labor progression” and “Cardiotocography during labor in healthy pregnant women with spontaneous labor” have limitations in their measurement. For the first, we considered the presence of monitoring records of labor progression (digital vaginal examination and auscultation of fetal heart rate), as these records allow assessing the well-being of a woman and her baby. However, we do not have information on the monitoring interval, for example, for cervical dilation, which should be done every 4 h [23]. We also did not identify the partogram model used and whether warning and action lines were used, which is currently not recommended [23]. For these reasons, it is possible that the adequacy of this practice is overestimated. Regarding the variable called “cardiotocography during labor in healthy pregnant women with spontaneous labor”, the main limitation is the lack of information regarding the type of use (whether or not the use is continuous), which may have overestimated inappropriate use. The hospitals included in this study were selected according to a convenience sample, and the results found cannot be extrapolated to the set of hospitals that are part of the PPA. However, the results are consistent with the literature on the subject in national and international publications. Finally, the small number of vaginal births assisted by nurses prevented the assessment of the adequacy of practices during childbirth, thus a gap in knowledge remains.

## Conclusions

The results of this study show an increase in the proportion of women with spontaneous or induced labor, with vaginal birth and with appropriate use of recommended practices in the “PPA model of care”. However, the proportion of women assisted by nurse-midwives during labor and vaginal birth did not differ between the two models of care, and the use of non-recommended practices is still high.

The PPA is a quality improvement project that is still in progress and the observed results suggest the need for improvements, especially the expansion of nursing participation in labor and childbirth care, the latter being practically nonexistent, and the increase in the use of recommended practices during labor and the reduction in the use of non-recommended practices.

With all these challenges still present, we consider that the PPA is a promising strategy to reverse the increase in the rate of cesarean sections, through an innovative and viable model of childbirth care, that can be applicable to other countries with excess CS rates [17].

Future studies should investigate how the low insertion of nurse-midwives affects the autonomy of these professionals in the care provided to women and babies, as well as how the PPA and similar quality improvement projects could strengthen strategies that support nurse-midwives and midwives and improve overall childbirth care outcomes.

## Data Availability

All data generated or analysed during this study are included in this published article.

## Abbreviations

ANS: National Supplementary Health Agency
CS: Cesarean section
PPA: Adequate Childbirth Project (Projeto Parto Adequado)
SoC: Standard of care model
WHO: World Health Organization

## References

[1] Patah LEM, Malik AM (2011). Modelos de assistência ao parto e taxa de cesárea em diferentes países. Rev Saúde Pública.

[2] Brasil. Ministério da Saúde. Secretaria de Atenção à Saúde. Humanização do parto e do nascimento. 1ª ed. Brasília: Ministério da Saúde; 2014.

[3] Diniz SG, Chacham AS (2004). “The Cut above” and “the cut below”: the abuse of caesareans and episiotomy in São Paulo, Brazil. Reprod Health Matters.

[4] Brasil. Diretrizes nacionais de assistência ao parto normal: versão resumida. 1ª ed. Brasília: Ministério da Saúde; 2017.

[5] Souza JP, Betran AP, Dumont A, de Mucio B, Gibbs Pickens CM, Deneux-Tharaux C (2015). A global reference for caesarean section rates (C-Model): a multicountry cross-sectional study. BJOG.

[6] Brasil. Diretrizes de atenção à gestante: a operação cesariana. 1ª ed. Brasília: Ministério da Saúde, 2015.

[7] OMS. Declaração da OMS sobre Taxas de Cesáreas. In: human reproduction programme. 2015. http://apps.who.int/iris/bitstream/handle/10665/161442/WHO_RHR_15.02_por.pdf;jsessionid=E62D51B0A616300075B18C58DF68A204?sequence=3. Accessed 10 Oct 2018.

[8] Domingues RMSM, Torres JA, Leal MC, Hartz ZMA (2019). Contextual factors in the analysis of the implementation of a multifaceted intervention in Brazilian private hospitals: initial reflections of the evaluative research "healthy birth". Anais do IHMT.

[9] Dias MAB, Domingues RMSM, Esteves-Pereira AP, Fonseca SC, Gama SGN, Theme Filha MM (2008). Trajetória das mulheres na definição pelo parto cesáreo: estudo de caso em duas unidades do sistema de saúde suplementar do estado do Rio de Janeiro. Ciência e Saúde Coletiva.

[10] Domingues RMSM, Dias MAB, Nakamura-Pereira M, Torres JA, d'Orsi E, Esteves-Pereira AP (2014). Process of decision-making regarding the mode of birth in Brazil: from the initial preference of women to the final mode of birth. Cad Saude Publica.

[11] Nascimento RRP, Arantes SL, Souza EDC, Contrera L, Sales APA (2015). Escolha do tipo de parto: fatores relatados por puérperas. Rev Gaucha Enferm.

[12] OMS. Prevenção e eliminação de abusos, desrespeito e maus-tratos durante o parto em instituições de saúde. In: human reproduction programme. 2014. https://apps.who.int/. Accessed 10 Jul 2020.

[13] Torres JA, Domingues RMSM, Sandall J, Hartz Z, Gama SGN, Theme Filha MM (2014). Cesariana e resultados neonatais em hospitais privados no Brasil: estudo comparativo de dois diferentes modelos de atenção perinatal. Cad Saude Publica.

[14] Sandall J, Soltani H, Gates S, Shennan A, Devane D (2016). Midwife-led continuity models versus other models of care for childbearing women. Cochrane Database Syst Rev.

[15] Gama SGN, Viellas EF, Torres JA, Bastos MH, Brüggemann OM, Theme Filha MM (2016). Labor and birth care by nurse with midwifery skills in Brazil. Reprod Health.

[16] Brasil. Parto Adequado. In: Agência Nacional de Saúde Suplementar. 2020. http://www.ans.gov.br/gestao-em-saude/parto-adequado. Accessed 14 Dec 2020.

[17] Borem P, Sanchez RC, Torres J, Delgado P, Petenate AJ, Peres D (2020). A quality improvement initiative to increase the frequency of vaginal delivery in Brazilian hospitals. Obstet Gynecol.

[18] Narchi NZ, Cruz EF, Gonçalves R (2013). O papel das obstetrizes e enfermeiras obstetras na promoção da maternidade segura no Brasil. Ciênc Saúde Colet.

[19] WHO (2013). Nursing and midwifery progress report 2008–2012.

[20] Vogt SE, Silva KS, Dias MAB (2014). Comparação de modelos de assistência ao parto em hospitais públicos. Rev Saude Publica.

[21] Torres JA, Leal MC, Domingues RMSM, Esteves-Pereira AP, Nakano AR, Gomes ML (2018). Evaluation of a quality improvement intervention for labour and birth care in Brazilian private hospitals: a protocol. Reprod Health.

[22] WHO. Care in normal birth: a practical guide. Universidade de Michigan: Maternal and Newborn Health/Safe Motherhood Unit, Family and Reproductive Health, World Health Organization; 1996. 10.1111/j.1523-536X.1997.00121.pp.x.

[23] WHO (2018). WHO recommendations: intrapartum care for a positive childbirth experience.

[24] Robson MS (2001). Can we reduce the caesarean section rate?. Best Pract Res Clin Obstet Gynaecol.

[25] Associação Brasileira de Empresas de Pesquisa. Critério Brasil 2020. 2019. http://www.abep.org/criterio-brasil. Acessed 01 Feb 2021.

[26] Alves CKA, Natal S, Felisberto E, Samico I. Interpretação e análise das informações: o uso de matrizes, critérios, indicadores e padrões. In: Samico I, Felisberto E, Figueiró AC, Frias PG, organizadores. Avaliação em saúde: bases conceituais e operacionais. Rio de Janeiro: MedBook; 2010.

[27] SPSS Inc. Released 2008. SPSS statistics for windows, version 17.0. Chicago: SPSS Inc.

[28] Leal MC, Bittencourt SA, Esteves-Pereira AP, Ayres BVS, Silva LBRAA, Thomaz EBAF (2019). Avanços na assistência ao parto no Brasil: resultados preliminares de dois estudos avaliativos. Cadernos de Saúde Pública.

[29] Birthplace in England Collaborative Group (2011). Perinatal and maternal outcomes by planned place of birth for healthy women with low risk pregnancies: the Birthplace in England national prospective cohort study. BMJ.

[30] Oliveira CF, Ribeiro AAV, Luquine Junior CD, Bortoli MC, Toma TS, Chapman EMG (2020). Barreiras à implementação de recomendações para assistência ao parto normal: revisão rápida de evidências. Rev Panam Salud Publica.

[31] Nakamura-Pereira M, Leal MC, Esteves-Pereira AP, Domingues RMSM, Torres JA, Dias MAB (2016). Use of Robson classification to assess cesarean section rate in Brazil: the role of source of payment for childbirth. Reprod Health.

[32] Vogt SE, Diniz SG, Tavares CM, Santos NCP, Schneck CA, Zorzam B (2011). Características da assistência ao trabalho de parto e parto em três modelos de atenção no SUS, no Município de Belo Horizonte, Minas Gerais. Brasil Cadernos de Saúde Pública.

[33] Gama SGN, Viellas EF, Medina ET, Angulo-Tuesta A, Silva CKRT, Silva SD, et al. Atenção ao parto por enfermeira obstétrica emmaternidades vinculadas à Rede Cegonha, Brasil—2017. Cien Saude Colet [periódico na internet]. 2020. http://www.cienciaesaudecoletiva.com.br/artigos/atencao-ao-parto-por-enfermeira-obstetrica-emmaternidades-vinculadas-a-rede-cegonha-brasil-2017/17730?id=17730. Accessed 09 Jan 2021. 10.1590/1413-81232021263.28482020 33729347

[34] Brasil. Ministério da Saúde. Portaria no 1.459, de 24 de junho de 2011. http://bvsms.saude.gov.br/bvs/saudelegis/gm/2011/prt1459_24_06_2011.html. Accessed 10 Feb 2021.

[35] Souza LV. Fontes para a história da ginecologia e obstetrícia no Brasil. História, Ciência, Saúde—Manguinhos. 2018. 10.1590/s0104-59702018000500011. 10.1590/S0104-59702018000500011 30624481

[36] . Ferreira LO. Negócio, política, ciência e vice-versa: uma história institucional do jornalismo médico brasileiro entre 1827 e 1843. História, Ciências, Saúde—Manguinhos; 2004. 10.1590/S0104-59702004000400005. 10.1590/s0104-59702004000400005 15446259

[37] Silva F, Nucci M, Nakano AR, Teixeira L (2019). “Parto ideal”: medicalização e construção de uma roteirização da assistência ao parto hospitalar no Brasil em meados do século XX. Saúde e Sociedade.

[38] Sena CD, Santos TCS, Carvalho CMF, Sá ACM, Paixão GPN (2012). Avanços e retrocessos da enfermagem obstétrica no Brasil. Revista de Enfermagem da UFSM.

[39] Progianti JM, Moreira NJMP, Prata JA, Vieira MLC, Almeida TA, Vargens OMC (2018). Precarização do trabalho da enfermeira obstétrica/job insecurity among obstetric nurses/Precarización del trabajo de la enfermera obstétrica. Rev enferm UERJ.

[40] Magluta C, Noronha MF, Gomes MAM, Aquino LA, Alves CA, Silva RS (2009). Estrutura de maternidades do Sistema Único de Saúde do Rio de Janeiro: desafio à qualidade do cuidado à saúde. Rev Bras Saude Mater Infant.

[41] Centro Latino-Americano de Perinatologia, Saúde da Mulher e Reprodutiva. Conjunto de ferramentas para o fortalecimento da obstetrícia nas Américas. Montividéu: CLAP/SMR; 2013.

[42] Homer CSE, Friberg IK, Dias MAB, ten Hoope-Bender P, Sandall J, Speciale AM (2014). The projected effect of scaling up midwifery. The Lancet.

[43] Barreto JOM, Bortoli MC, Luquine CD, Oliveira CF, Toma TS, Ribeiro AAV (2020). Implementation of the National Childbirth Guidelines in Brazil: barriers and strategies. Rev Panam Salud Publica.

[44] OMS. Relatório Mundial da Saúde 2005: para que todas as mães e crianças contem. In: Publicações da OMSGenebra, Suíça.:. Organização Mundial da Saúde. 2005. https://www.who.int/whr/2005/media_centre/overview_pt.pdf?ua=1. Accessed 12 Aug 2020.

[45] Oliveira APC, Ventura CAA, Galante ML, Padilla M, Cunha A, Mendes IAC (2021). The current state of obstetric nursing in Brazil. Rev Latino-Am Enfermagem.

[46] Wagner M (2001). Fish can’t see water: the need to humanize birth. Int J Gynaecol Obstet.

[47] Victora CG, Aquino EMMLL, Leal MC, Monteiro CA, Barros FC, Szwarcwald CL (2011). Maternal and child health in Brazil: progress and challenges. Lancet.

[48] Victora CG, Barreto ML, Leal MC, Monteiro CA, Schmidt MI, Paim JS (2011). Health conditions and health-policy innovations in Brazil: the way forward. The Lancet.

